# Increased osteoprotegerin level is associated with impaired cardiovagal modulation in type-2 diabetic patients treated with oral antidiabetic drugs

**DOI:** 10.1186/s12872-020-01729-1

**Published:** 2020-10-20

**Authors:** M. R. Jasmine, Nivedita Nanda, Jayaprakash Sahoo, S. Velkumary, G. K. Pal

**Affiliations:** 1 Department of Biochemistry, Jawaharlal Institute of Postgraduate Medical Education and Research (JIPMER), Puducherry, 605 006 India; 2 Department of Endocrinology, Jawaharlal Institute of Postgraduate Medical Education and Research, Puducherry, India; 3 Department of Physiology, Jawaharlal Institute of Postgraduate Medical Education and Research, Puducherry, India

**Keywords:** Type-2 diabetes mellitus, Sympathovagal imbalance, Cardiovagal modulation, Heart rate variability, Osteoprotegerin, Cardiometabolic risks

## Abstract

**Background:**

An increased osteoprotegerin (OPG) level has been reported in both type-2 diabetes mellitus (T2DM) and cardiovascular diease (CVD) that are linked to sympathovagal imbalance (SVI). We explored the link of osteoprotegerin with cardiovagal modulation in T2DM.

**Methods:**

We assessed fasting serum OPG, high-sensitive C-reactive protein (hsCRP), glucose, insulin and lipid profile in patients having T2DM receiving oral antidiabetic drugs (OAD) (n = 42) compared with age, gender and body composition-matched healthy participants without diabetes (n = 42). Rate pressure product (RPP), spectral indices of heart rate variability (HRV) and body composition were recorded in both the groups. Association of HOMA-IR and OPG with various parameters were assessed.

**Results:**

Osteoprotegerin, HOMA-IR, hsCRP, coronary lipid risk factor were significantly increased, markers of cardiovagal modulation (TP, SDNN, RMSSD) were considerably decreased, ratio of low-frequency to high-frequency (LH-HF ratio), the indicator of SVI, and RPP, the marker of myocardial work stress were significantly higher in patients with diabetes, suggesting an overall elevated CVD risks in them. HOMA-IR was correlated with RMSSD, lipid risk factors and OPG. Rise in OPG was correlated with decreased cardiovagal modulation in patients with diabetes. There was significant contribution of OPG in decreasing TP, suggesting impaired cardiovagal modulation.

**Conclusion:**

T2DM patients receiving OAD had higher cardiometabolic risks compared to age, gender and body composition-matched healthy individuals. Increased level of OPG is linked to decreased cardiovagal modulation in T2DM patients.

## Background

A recent report with pooled data from 751 studies consisting of 4,372,000 adults from 146 countries has projected that the total number of diabetic adults worldwide has increased from 108 million in 1980 to 422 million by 2014 and age-standardized diabetes prevalence has increased from 4.3 to 9.0% in men and 5.0 to 7.9% in women on a global scale [1]. India alone harbours 62 million of diabetics, making the country the diabetic capital of the world [2]. In spite of early diagnosis and treatment, type-2 diabetes mellitus (T2DM) is the leading cause of morbidity and mortality in India, and cardiovascular diseases (CVD) are the common complications of diabetes [3]. Accelerated atherosclerosis, hypertension, inflammation, oxidative stress and obesity are several traditional risk factors for CVD in diabetes that are linked to poor glycemic control in spite of patients with diabetes getting regular treatment for the disease [4, 5].

Osteoprotegerin (OPG) is a glycoprotein, secreted mainly from vascular smooth muscle cells and adipocytes [5]. Though in physiological concentration it mainly prevents arterial calcification, higher plasma levels of OPG has been reported to contribute to the progression of vascular dysfunction and inflammation and elevated OPG is proposed as a marker of progressive atherosclerosis and CVD [6]. Though, elevated circulating levels of OPG have been reported in T2DM, particularly in the presence of microvascular complications [7] and peripheral neuropathy [8–10], the role of OPG in autonomic neuropathy in diabetes has not been adequately studied. A recent report did not demonstrate the association of OPG and osteopontin with cardiovascular autonomic function in T2DM patients [11]. Further, the reports on OPG level in conditions of insulin resistance (IR) are conflicting, especially in the presence of obesity [12, 13]. Therefore, in the present study we have assessed the link of OPG with autonomic dysfunctions in T2DM patients.

Autonomic imbalance representing hyperactive sympathetic system and hypoactive parasympathetic system is known as sympathovagal imbalance [14, 15]. Sympathovagal imbalance acts as a physiological contributor to metabolic dyshomeostasis [16] and cardiovascular (CV) risks in various clinical conditions [17, 18]. Sympathovagal imbalance increases CV risk attributed by insulin resistance, inflammation, oxidative stress and dyslipidemia [19]. As dysfunction of the autonomic nervous system (ANS) can predict CV risk and sudden death in T2DM [20], its early detection can improve the prognosis and reduce adverse cardiac events in patients with diabetes. Analysis of heart rate variability (HRV) is a non-invasive and sensitive method to assess sympathovagal imbalance at an early stage of autonomic dysfunction, qualifying it as an early marker of CV risk [21]. Decreased vagal modulation of cardiac function has recently been established as CV risk [22, 23]. In HRV analysis, the successive RR interval differences are produced solely by the parasympathetic system, which is quantified mainly by calculating the root mean square of the sum of successive RR interval differences (RMSSD) [21]. Standard deviation of the inter-beat interval of normal sinus beats (SDNN), another time-domain index of HRV, reflecting the effect of vagal drive on heart, is used for stratification of CV risk [22]. It has been reported that just one standard deviation of decrease in SDNN increases the odds of developing metabolic syndrome by 43% [16]. Therefore, RMSSD and SDNN are considered as important measures of cardiovagal modulation [24]. However, till date the status of RMSSD and SDNN in patients with diabetes on oral antidiabetic drugs has not been fully elucidated.

Body mass index (BMI) and body fat composition are known to contribute to vagal modulation of heart function [25, 26]. To best of our knowledge, no studies have been conducted yet to assess the link of OPG to cardiovagal modulation in Indian population, especially in the assessment of CV risks in patients with T2DM. Therefore, in the present study, we have assessed the link of OPG to cardiovagal modulation in BMI and body-composition matched patients with diabetes and participants without diabetes.

## Methods

### Study design

This was a case–control study conducted in the department of Biochemistry, Physiology and Endocrinology at Jawaharlal Institute of Postgraduate Medical Education and Research (JIPMER), Puducherry, India.

### Standard protocol approvals and sample size calculation

Approval was obtained from the Institute Research Committee and Institute Ethics Committee for conducting the study. The sample size was estimated using the statistical formula for comparing means with equal variance. The minimum expected difference in the level of OPG between the groups is 1 pM/L with a standard deviation of 1.6 pM/L [12] and the sample size estimated at 5% level of significance and 80% power. The minimum sample size estimated for this study was 40 in each group.

### Participants, grouping of the participants and inclusion and exclusion criteria

The case (study group) included T2DM patients on treatment with oral antidiabetic drugs (OAD) and controls (control group) were healthy participants without diabetes.

#### Study group

Patients with diabetes on OAD were contacted from Endocrinology Outpatient Department. Patients receiving only metformin and glimepiride for more than a year were recruited (n = 42) to the study group. To ensure uniformity of antidiabetic treatment among the diabetic patients and to exclude influence of medicines other than these two drugs on autonomic and metabolic functions, the patients receiving only the combination of metformin and glimepiride were selected for the study.

Patients on insulin therapy, receiving OAD other than metformin and glimepiride, age above 60 years and BMI more than 35 kg/m^2^, were excluded from the study. Individuals with the history of smoking, alcoholism, ischemic heart disease, renal disorder (creatinine more than 1.5 mg%), inflammatory diseases, morbid obesity and severe chronic illness, with features of acute infections and patients on glucocorticoid or immuno-suppressive therapy, were excluded from study group.

#### Control group

Healthy volunteers of age and gender similar to the study group participants were included as control. Participants with habit of smoking and alcoholism, practising yoga or regular sports activities, with recent history of infection or acute illness, and pregnant or lactating women, were excluded from the control group. Forty-two participants who were matched for BMI and body composition with study group participants were recruited for the study.
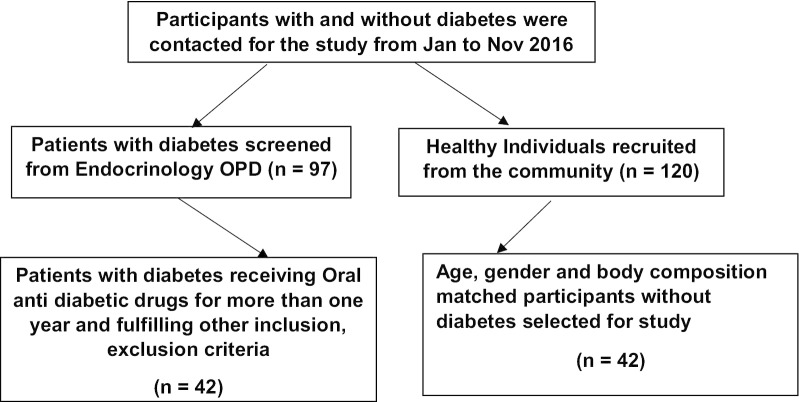


### Consent and basal recordings

The study protocol was explained to the patients with diabetes and participants without diabetes in their vernacular and written informed consent was obtained from all the participants before recording of the study parameters. Basal demographic and anthropometric parameters of all the participants such as age, body weight, body mass index (BMI), blood pressure (BP), basal heart rate (BHR) were recorded, and the personal history was noted using a structured data-sheet.

### Brief procedure

Participants were asked to report to Biochemistry department following an overnight fast for collection of venous blood sample, following which they were escorted to Physiology Obesity Research Lab for the recording of body fat composition and heart rate variability (HRV). The serum was separated and made into aliquots for storage at − 40 °C for further biochemical analysis, after estimating fasting glucose and lipid profile as routine investigations in clinical biochemistry lab. For all the participants, OPG, insulin, HOMA-IR and hsCRP were assayed from the stored sera using commercial ELISA kits.

#### Body composition analysis

Body fat composition was assessed by using Bodystat Quadscan 4000 (Bodystat, Douglas, UK). The Quadscan works on the principle of bioelectrical impedance using a multi-frequency-tetra polar technique. The main parameters recorded by Quadscan are body fat mass %, body fat content, lean body mass, body fat mass index (BFMI) and body fat-free mass index (FFMI).

#### Biochemical parameters

Fasting serum glucose, lipid profile (triglycerides, total cholesterol, HDL-cholesterol, LDL-cholesterol & VLDL-cholesterol) were measured using commercial kits adapted to clinical chemistry autoanalyzer (Olympus 400, Beckman Coulter, Orlando, FL, USA).

Coronary lipid risk factors of cardiovascular disease were calculated from lipid profile values as described before [27]. Osteoprotegerin was analysed using ELISA kit (RayBio®, M/S GenxBio health science, Delhi, India) following manufacturer's instructions. Insulin (DIAsource Immunoassay S.A, M/S Mouli diagnostics, Puducherry) and high-sensitive C-reactive protein (hsCRP) were analysed using ELISA kits (Cal biotech, M/S Bio-diagnosis, Chennai), following manufacturer’s instructions. Homeostatic model assessment of insulin resistance (HOMA-IR) was calculated using the formula fasting glucose × Insulin/22.5.

#### Heart rate variability (HRV) analysis

HRV was recorded and analyzed as per the recommendation of Task Force on HRV [24] and as practiced in our AFT Lab [23], by continuous ECG recordings for 10 min using BIOPAC MP-100 data-acquisition system and Acknowledge software version 3.8.2 (BIOPAC Inc, USA). Time-domain indices of HRV such as SDNN and RMSSD, and frequency-domain indices of HRV such as total power (TP), low-frequency (LF) and high-frequency (HF) power and LF/HF ratio were calculated by the software.

### Statistical analysis of data

All statistical analyses were done using SPSS software package version 13 (Chicago, IL, USA). Data were analysed by Kolmogrov–Smironoff test for the normality. All data were expressed as mean and SD. Comparison between control and test group was done by Student’s *t* test for continuous parametric data and by Mann Whitney U test for nonparametric data. The strength of correlation among parameters was analysed by Spearman’s rank correlation analysis for parameters with skewed distribution and by Pearson’s correlation for parameters with normal distribution. The association of TP with OPG was assessed by linear regression analysis.

## Results

Forty-two diabetes mellitus patients and forty-two participants without diabetes as control were recruited in the study with a mean age of 45.76 ± 6.88 years and 43.04 ± 6.21 years respectively. The BMI was 27.03 ± 3.93 and 25.59 ± 3.27 for study group and control group respectively. There was significant increase in basal heart rate, SBP, DBP and RPP in patients with diabetes compared to participants without diabetes (Table 1). TP was significantly less (*p* < 0.001), LH-HF ratio was significantly more (*p* < 0.05) and time domain indices of HRV such as RMSSD and SDNN were significantly lower (*p* < 0.001) in patients with diabetes (Table 1).

**Table 1:** Comparison of age, anthropometric indices, body fat composition, basal heart rate, blood pressure and heart rate variability (HRV) indices between control group (participants without diabetes) and diabetes group (patients with diabetes)

	Control group	Diabetes group
	(n = 42)	(n = 42)
General parameters		
Female:Male	27:15	27:15
Age (years)	43.04 ± 6.21	45.76 ± 6.88
BMI (Kg/m^2^)	25.59 ± 3.27	27.03 ± 3.93
Waist/hip ratio	0.87 ± 0.05	0.90 ± 0.06
Body fat composition		
Body fat (%)	35.95 ± 7.65	37.71 ± 8.18
BFMI	9.30 ± 2.81	10.32 ± 3.17
LBM (%)	64.04 ± 7.65	62.28 ± 8.18
FFMI	16.29 ± 2.13	16.70 ± 2.55
BHR and BP parameters		
BHR (beats per min)	70.19 ± 8.93	82.21 ± 10.99***
SBP (mmHg)	116.40 ± 10.45	123.88 ± 10.51**
DBP (mmHg)	73.66 ± 9.13	78.61 ± 8.95**
RPP (mmHg/min)	82.07 ± 10.19	101.76 ± 15.46***
HRV indices		
TP (ms^2^)	566.23 ± 311.37	285.35 ± 197.57***
LFnu	48.82 ± 16.31	56.85 ± 19.47
HFnu	61.40 ± 24.79	43.06 ± 19.50
LF/HF	1.23 ± 0.99	2.08 ± 2.07*
SDNN (ms)	25.39 ± 9.92	17.45 ± 6.92***
RMSSD (ms)	25.01 ± 9.04	16.94 ± 8.03***

The values are expressed as Mean ± SD

*BMI* body mass index, *BFMI* body fat mass index, *LBM* lean body mass, *FFMI* free fat mass index, *BHR* basal heart rate, *SBP* systolic blood pressure, *DBP* diastolic blood pressure, *RPP* rate pressure product, *RPP* rate pressure product = SBP × HR/100, *TP* total power of HRV, *LFnu* normalized low-frequency power of HRV, *HFnu* normalized high-frequency power of HRV, *SDNN* standard deviation of normal to normal interval, *RMSSD* square root of the mean of the sum of the squares of the differences between adjacent NN intervals

^*^p < 0.05; **p < 0.01;***p < 0.001; by Student’s unpaired ‘*t*’ test

FSG, insulin and HOMA-IR were significantly increased in diabetes group compared to control group (Table 2). Though there was no significant difference in TC and HDL, the LDL was significantly decreased (*p* < 0.01) and TG and VLDL were significantly increased (*p* < 0.001) in the diabetes group. Atherogenic index of plasma (AIP) was significantly high in diabetes group compared to control group (*p* < 0.05). The hsCRP (*p* < 0.05) and OPG (*p* < 0.001) were significantly increased in diabetes group compared to control group (Table 2). The TG, lipid risk factors [TG/HDL-C, TC/HDL-C, LDL-C/HDL-C and AIP] were positively correlated with HOMA-IR in diabetes group and there was no correlation for any of these parameters in control group (Table 3). TP (r = − 0.357, *p* = 0.020), SDNN (r = − 0.345, *p* = 0.029) and RMSSD (r = − 0.333, *p* = 0.036) were significantly correlated with OPG in diabetes group (Table 4). The significant contribution of OPG to TP (β = − 0.357, *p* = 0.020) was demonstrated by linear regression analysis (Table 5).

**Table 2:** Comparison of glycaemic parameters, lipid profile and lipid risk factors and other biochemical parameters between control group (participants without diabetes) and diabetes group (patients with diabetes)

	Control Group	Diabetes Group
	(n = 42)	(n = 42)
Glycemic parameters		
FSG (mg/dL)	84.95 ± 14.86	162.74 ± 75.27***
Insulin (μU/mL)	3.31 ± 1.27	6.76 ± 3.96 ^ΨΨΨ^
HOMA-IR	0.72 ± 0.41	3.17 ± 3.04 ^ΨΨΨ^
Lipid profile		
TC (mg/dL)	193.02 ± 38.86	175.93 ± 35.45
HDL-C (mg/dL)	42.88 ± 9.31	43.54 ± 9.87
LDL-C (mg/dL)	131.10 ± 31.53	103.60 ± 38.35**
TG (mg/dL)	113.21 ± 84.13	144.45 ± 69.27 ^ΨΨΨ^
VLDL-C (mg/dL)	22.64 ± 16.82	28.88 ± 13.86 ^ΨΨΨ^
Lipid risk factors		
Non HDL-C (mg/dL)	150.14 ± 38.26	132.38 ± 36.23
TG/HDL-C	2.78 ± 2.11	3.78 ± 2.92 ^Ψ^
TC/HDL-C	4.67 ± 1.21	4.26 ± 1.33
LDL-C/HDL-C	3.18 ± 0.95	2.52 ± 1.18*
AIP	0.35 ± 0.27	0.48 ± 0.27*
Inflammatory markers		
hsCRP (mg/L)	0.72 ± 0.38	1.09 ± 1.31^Ψ^
Osteoprotegerin (pg/mL)	131.20 ± 73.66	225.54 ± 128.78 ***

The values are expressed as Mean ± SD

*FSG* fasting serum glucose, *HOMA-IR* homeostatic model assessment of insulin resistance, *TC* total choleterol, *HDL-C* high density cholesterol, *LDL-C* low density cholesterol, *TG* triglyceride, *VLDL-C* very low density cholesterol, *Non HDL-C* non HDL cholesterol, *AIP* atherogenic index of plasma = log_10_[TG/HDL-C], *hsCRP* high-sensitive C-reactive protien

^*^p < 0.05; **p < 0.01;***p < 0.001 by Student’s unpaired ‘*t*’ test

^Ψ^*p* < 0.05; ^ΨΨΨ^*p* < 0.001 by Mann Whitney U test

**Table 3:** Spearman rank correlation analysis of serum HOMA-IR with various parameters between control group (participants without diabetes) and diabetes group (patients with diabetes)

Parameters	Control group		Diabetes group	
	*r*	*p*	*r*	*p*
RPP	0.064	0.690	− 0.118	0.456
TP	0.200	0.229	− 0.146	0.358
SDNN	0.172	0.301	− 0.210	0.193
RMSSD	0.011	0.949	− 0.362	0.022
TG	0.187	0.241	0.379	0.013
TG/HDL-C	0.222	0.163	0.427	0.005
TC/HDL-C	− 0.024	0.881	0.510	0.001
LDL-C/HDL-C	0.016	0.919	0.440	0.004
AIP	0.222	0.163	0.427	0.005
OPG	− 0.251	0.145	0.323	0.037

*RPP* rate pressure product = SBP × HR/100, *TP* total power of HRV, *SDNN* standard deviation of normal to normal interval, *RMSSD* square root of the mean of the sum of the squares of the differences between adjacent NN intervals, *TG *triglyceride, *HDL-C* high density cholesterol, *TC* total choleterol, *LDL-C* low density cholesterol, *AIP* atherogenic index of plasma = log_10_[TG/ HDL-C]

*p* < 0.05 was considered significant

**Table 4:** Pearson correlation analysis of serum OPG with HRV between control group (participants without diabetes) and diabetes group (patients with diabetes)

Parameters	Control group		Diabetes group	
	*r*	*p*	*r*	*p*
TP	0.206	0.234	− 0.357	0.020
SDNN	0.135	0.439	− 0.345	0.029
RMSSD	0.246	0.145	− 0.333	0.036

*TP* total power of HRV, *SDNN* standard deviation of normal to normal interval, *RMSSD* square root of the mean of the sum of the squares of the differences between adjacent NN intervals

*p* < 0.05 was considered significant

**Table 5:** Linear regression analysis to assess the independent association of TP (as dependant variable) with OPG (as independent variables) in diabetes group, adjusted for glycemic status

Independent variable	Standardized regression coefficient beta	95% confidence interval		*p* value
		Upper limit	Lower limit	
OPG	− 0.357	− 1.001	− 0.089	0.020

The *p* value < 0.05 was considered significant

*TP* total power of heart rate variability, *OPG* osteoprotegerin

## Discussion

**Fig. 1:**
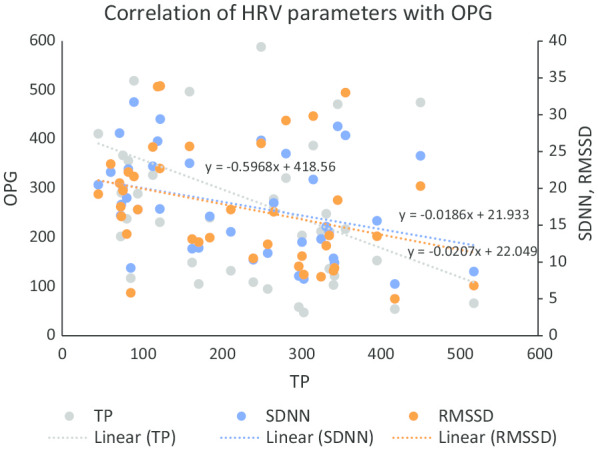
Scatter plot showing correlation of serum OPG with HRV parameters (TP, SDNN and RMSSD) in participants with diabetes. *OPG* osteoprotegerin, *TP* total power of HRV, *SDNN* standard deviation of normal to normal interval, *RMSSD* square root of the mean of the sum of the squares of the differences between adjacent NN intervals

In the present study, OPG was significantly elevated in patients with diabetes receiving metformin and glimepiride for more than one year, compared with the participants without diabetes (Table 2). Though there are earlier reports of increased OPG in T2DM [7–10], the reports on status of OPG in diabetic patients receiving the sole combination of metformin and glimepiride are scanty. Previous reports in patients receiving OAD suggests that these drugs have varying effects on circulating level of OPG in type-2 diabetic patients [28, 29]. Metformin therapy in a group of patients of non-alcoholic fatty-liver disease resulted in decreased serum OPG in just 4 months [30]. However, another study comparing the effects between metformin and pioglitazone exhibited that metformin had no effect on serum OPG level despite 6 months treatment, while pioglitazone given alone successfully decreased OPG level [28]. In another report, glimepiride monotherapy had significant rise in OPG in patients with diabetes compared to participants without diabetes [29]. Atorvastatin is reported to be prescribed frequently in diabetes and use of atorvastatin has been observed to decrease serum OPG level significantly [31]. In the present study, 45% (19/42) patients on metformin and glimepiride treatment also had atorvastatin therapy. Thus, despite combination therapy of these OAD and statin, there was significant rise in circulating OPG, indicating that OPG remains elevated inspite of adequate treatments in these patients (Fig. 1).

Among the HRV indices, TP, SDNN and RMSSD represent cardiac parasympathetic drive [24, 32]. There was significant decrease in TP, SDNN and RMSSD in diabetes group (*p* < 0.001) compared to the control group (Table 1), indicating decreased cardiovagal modulation in these patients with diabetes. Further, OPG was significantly correlated with TP, RMSSD and SDNN (Table 4) suggesting that decreased cardiovagal modulation could be linked to increased OPG. The TP of HRV represents the magnitude of variability of heart rate and is considered as the most important marker of vagal potency of cardiac modulation [32]. In the present study, significant association of OPG with TP in patients with diabetes as demonstrated by linear regression analysis (Table 5), indicates the possible contribution of OPG to decreased cardiovagal modulation in T2DM patients on OAD.

Though endothelial cells of blood vessels are the major source of OPG, it is also secreted by adipocytes contributing to its circulating level. Obesity is known to influence the cardiovagal modulation [26]. Therefore, in the present study, we have recruited BMI and body-composition matched participants without diabetes, to nullify the influence of adiposity on OPG. OPG is a component of tumor necrosis factor α (TNF-α) receptor superfamily. It is secreted from adipocytes and vascular smooth muscle cells, and physiologically it is involved in bone metabolism [33]. OPG has been reported to control the progression of vascular dysfunction in DM [34]. Increased OPG production is proposed to induce endothelial inflammation that promotes atherosclerotic lesions [35]. Recent evidences suggest that OPG is independently associated with progressive atherosclerosis and incident CVD [34, 35]. Earlier reports have shown that OPG and insulin resistance are linked to obesity [12, 13], and sympathovagal imbalance has also been reported in obesity [25, 26]. Therefore, in the present study we have assessed the parameters of sympathovagal imbalance of HRV.

Increase in OPG in metabolic syndrome (MS) has been reported to trigger adipose tissue proinflammatory changes [36]. Moreover, a proinflammatory milieu, hyperglycemia and hyperinsulinemia that characterize MS, lead to further increase in secretion of OPG by endothelial cells, and therefore, OPG has been proposed as a potential biomarker of MS [37]. OPG has been demonstrated to be the independent predictor of MS even after adjusting for age, gender, ethnicity, glucose and microvascular complications [9] and consequently, OPG has been suggested as a target of therapy in T2DM [38]. All these metabolic alterations such as insulin resistance, oxidative stress and retrograde inflammation have been reported to contribute to autonomic dysfunctions in diabetes [19, 20]. Further, there are reports of association between higher OPG levels and peripheral neuropathy in diabetes [8, 10]. Also, there is indication of a possible link of OPG with autonomic dysfunctions [11]. Therefore, the possible mechanism of OPG causing decreased cardiovagal modulation in diabetic subjects in the present study could be the metabolic alterations, especially the proinflammatory milieu in these patients, as hsCRP the marker of inflammation was significantly more in patients compared to controls.

Significant increase in LF/HF ratio in patients with diabetes compared to participants without diabetes indicate increased sympathetic drive and decreased vagal drive to the heart in patients with diabetes, as LFnu is the marker of cardiac sympathetic modulation and HFnu is the indicator of cardiac parasympathetic modulation [24]. In our study, patients with diabetes had significantly increased LF/HF ratio compared to the participants without diabetes, suggesting the presence of considerable sympathovagal imbalance with increased sympathetic drive in diabetic patients. These findings are in agreement with our previous reports of autonomic imbalance and decreased parasympathetic modulation in T2DM [39, 40], which also corroborates with the report by others [38]. However, in previous report by Sucharita et al. [41], the authors did not measure SDNN and RMSSD, the primary HRV indices of cardiovagal modulation.

Significant rise in resting or basal heart rate (BHR) in study group participants compared to control group participants (Table 1) suggests decreased vagal tone in patients with diabetes (Table 1), as heart rate in resting state is the marker of vagal tone [42]. Further, resting tachycardia in these patients with diabetes could be a CV risk in them, as BHR more than 75 per min per se has been reported as an established CV risk [42]. The considerable rise in SBP and DBP in diabetes group compared to control group (Table 1) suggests a higher sympathetic tone in patients with diabetes, as basal increase in blood pressure represents increased sympathetic vasoconstrictor tone [43]. In the present study, SBP of 59% (59/42) of the diabetic population was in the prehypertensive range (SBP between 120 and 139 mmHg), though they were not on any antihypertensive medications including beta blockers. Rate pressure product (RPP) was significantly elevated in these patients (Table 1). RPP is a marker of increased myocardial oxygen demand and increased RPP is considered as a physiological indicator of CV risk. It has been reported that increased RPP in hypertensive patients is an important CV risk [44]. Thus, in the present study, the resting tachycardia, increased BP and RPP indicate increased CV risks in patients with diabetes receiving OAD.

The measurement of HRV is a non-invasive and sensitive method of assessing sympathovagal drives [24] and reduction in TP of HRV is a sign of poor CV health [32]. Time-domain indices of HRV such as RMSSD and SDNN quantify the extent of variation in heart rate and the beat to beat vagal control of heart functions. RMSSD in particular estimates the impact of vagus mediated changes on HRV [32]. In the present study, significantly reduced TP, SDNN and RMSSD along with resting tachycardia and increase in blood pressure in the prehypertension range increases risk of cardiac morbidity due to decreased cardiovagal modulation. Decreased vagal tone has been reported to contribute to poor CV health [45]. In the present study, RMSSD was correlated with the HOMA-IR in patients with diabetes, but not in the control group participants (Table 3), indicating that the decreased cardiovagal modulation could be associated with the glycemic status in these patients. TP is the marker of parasympathetic strength of cardiac autonomic control [25] and a sensitive indicator of CV risk [32]. Therefore, to assess the independent association of OPG with TP of HRV, the glycemic status was adjusted in statistical analysis, and significant correlation was observed between OPG and TP in diabetes group (Table 5). These findings indicate that increase in OPG independently contributes to decreased cardiovagal modulation in T2DM patients receiving metformin and glimepiride therapy.

In the present study, the diabetic patients were on oral metformin and glimepiride therapy. Earlier it was reported that metformin by its direct central nervous system site of action produces acute sympathoinhibitory effects that decreases arterial pressure, heart rate, and efferent renal sympathetic nerve activity [46]. Further, it has been demonstrated that metformin treatment might be useful for improving cardiac sympathovagal balance in obese type 2 diabetic patients [47]. Thus, metformin might have influenced autonomic functions in diabetic patients in the present study and this could be a limitation of the study. Nevertheless, inspite of metformin therapy, which is known to inhibit sympathetic activity and improve sympathovagal balance in type 2 diabetic patients, in the present study we found increased sympathetic drive and considerable sympathovagal imbalance in diabetic patients. Thus, it appears that sympathovagal imbalance could have been more severe in these patients, had they not been received metformin therapy. Therefore, future studies may be planned to assess the role of OPG on cardiovagal modulation in diabetic patients not receiving metformin therapy.

CVDs are common in T2DM patients [4]. Several metabolic abnormalities such as hyperlipidemia, insulin resistance, inflammation and oxidative stress cross-link with each other to intensify the risk of CVD in diabetes [4]. In the present study, though there was no significant alteration in total cholesterol and HDL cholesterol among the two groups, LDL cholesterol was significantly low and triglyceride and VLDL were considerably high in study group (Table 2). Nineteen out of 42 participants received atorvastatin (10 mg/day), which might have contributed to the decrease in LDL cholesterol in the diabetes group. Our findings are in conformity with the report of Saely et al. [48], in which a larger cohort of T2DM patients with hypertension had lower LDL cholesterol compared to participants without diabetes. Further, in the present study, there was significantly high atherogenic index of plasma (AIP) in patients with diabetes (Table 2). Hypertriglyceridemia and all lipid risk factors for CVD were significantly correlated with increased level of HOMA-IR in T2DM patients (Table 3), indicating that atherogenic lipid profile in these patients with diabetes could be associated with the degree of insulin resistance. To best of our knowledge, this is the first report of correlation of HRV indices (TP, RMSSD, SDNN), triglyceride and atherogenic lipid risk factors with HOMA-IR in BMI- and body composition-matched participants from Indian subcontinent.

Another important issue in the present study is the considerable difference in heart rate between the controls and the patients. As heart rate has been reported to affect HRV strongly, the significantly more heart rate in diabetic patients might have influenced their HRV indices. To eliminate the influence of heart rate on HRV, recently the heart rate corrected HRV has been used as a better marker of autonomic functions in 10-s HRV recording [49]. However, it has also been investigated if the heart rate variability should be “corrected” for heart rate, as heart rate per se is a major determinant of the quantum of variability in HRV analysis; though it has been generally agreed upon that both adjusted and unadjusted HRV parameters can be used in the risk assessment studies [50]. Nonetheless, a larger sample size is needed for HRV to be adjusted for heart rate, statistically. Therefore, HRV adjusted for heart rate could not be calculated in the present study with the available sample size.

Due to the strict matching of participants with body composition and BMI, our sample size was modest, which is a limitation of the study. Nevertheless, it demonstrates the association of OPG level with decreased cardiovagal modulation independent of the effect of BMI, body composition and glycemic status. Thus, OPG could be used as a marker of cardiovagal modulation in these patients. Findings of the present study indicate that despite receiving oral antidiabetic drugs, the patients had subtle autonomic neuropathy in the form of decreased cardiac vagal drive. In developing countries like India, the management of chronic diseases like diabetes is never satisfactory, and therefore there is increased prevalence of CV risks in chronic diabetic patients. In our study, OPG emerged as an independent predictor of impaired cardiovagal modulation despite these patients being maintained on standard oral antidiabetic therapy. Therefore, periodic screening of circulating OPG apart from assessment of glycemic status can help the clinicians monitor the CV risks in patients with diabetes. OPG could be used for risk stratification of cardiovagal modulation in these patients. The report of the present study may encourage researchers and clinicians to take up future studies to assess the role of yoga and exercise that are known to improve cardiovagal modulation and improve sympathovagal balance [51], for better management of these patients in addition to the conventional treatment by oral antidiabetic drugs.

## Conclusion

Results of the present study indicate that despite regular treatment by oral antidiabetic agents, T2DM patients have higher cardiometabolic risks compared to their age, gender and body composition-matched participants without diabetes. The CV risks in these patients could be due to decreased vagal modulation of CV functions, attributed by insulin resistance, increased lipid risk factors, and retrograde inflammation. Osteoprotegerin could possibly be linked to the decreased cardiovagal modulation in these chronic diabetic patients.

## Data Availability

The datasets analysed in this study are available with the corresponding author, which can be obtained on reasonable request.

## Abbreviations

T2DM: Type-2 diabetes mellitus
CVD: Cardiovascular disease
OPG: Osteoprotegerin
hsCRP: High-sensitive C-reactive protein
OAD: Oral ant-diabetic drugs
HRV: Heart rate variability
AIP: Atherogenic index of plasma
TP: Total power
LF: Low-frequency
HF: High-frequency power
LH/HF: Low-frequency to high-frequency ratio
RPP: Rate pressure product
IR: Insulin resistance
SVI: Sympathovagal imbalance
ANS: Autonomic nervous system
BHR: Basal heart rate
BFMI: Body fat mass index
FFMI: Body fat-free mass index
